# A Metabonomic Analysis of Serum from Rats Treated with Ricinine Using Ultra Performance Liquid Chromatography Coupled with Mass Spectrometry

**DOI:** 10.1371/journal.pone.0090416

**Published:** 2014-03-11

**Authors:** Jing Peng, Shuang Cai, Lin Wang, Nan Zhao, Ting-jian Zhang, Zai-xing Chen, Fan-hao Meng

**Affiliations:** 1 School of Pharmacy, China Medical University, Shenyang, Liaoning, China; 2 Department of Pharmacy, The First Affiliated Hospital of China Medical University, Shenyang, Liaoning, China; Mayo Clinic, United States of America

## Abstract

A metabonomic approach based on ultra performance liquid chromatography coupled with mass spectrometry (UPLC/MS) was used to study the hepatotoxicity of ricinine in rats. Potential biomarkers of ricinine toxicity and toxicological mechanism were analyzed by serum metabonomic method. The significant differences in the metabolic profiling of the control and treated rats were clear by using the principal components analysis (PCA) of the chromatographic data. Significant changes of metabolite biomarkers like phenylalanine, tryptophan, cholic acid, LPC and PC were detected in the serum. These biochemical changes were related to the metabolic disorders in amino acids and phospholipids. This research indicates that UPLC/MS-based metabonomic analysis of serum samples can be used to predict the hepatotoxicity and further understand the toxicological mechanism induced by ricinine. This work shows that metabonomics method is a valuable tool in drug mechanism study.

## Introduction

*Ricinus communis*, a large red and green leaved castor bean plant, can be found in tropical and subtropical climates throughout the world, which has the therapeutic effect on considered anodyne, antidote, bactericide, cathartic, expectorant, insecticide, et al. The castor seed contains about 40% oil, 1–5% ricin, and 0.3–0.8% ricinine [1]–[5].

Ricinine (Nr- methyl-3-cyano-4-methoxy-2-pyridone) is a toxic alkaloid that is derived from the leaves and seeds of the castor bean plant [6], [7]. Ricinine may cause vomiting and various other toxic reactions, including liver and kidney damage, convulsions, hypotension, and death. There has been no report to demonstrate the toxicity mechanism of ricinine. Meanwhile, little is known about the changes in the whole metabolites of the organism after treatment with ricinine.

Metabonomics based on the analysis of entire pattern of low molecular weight compounds rather than focusing on individual metabolites, indicates a general procedure that gives information on whole organism functional integrity over time following exposition of a perturbation. It could be defined as an attempt to measure the variation in the metabolites that are presented within cell, biofluid or tissue during the genetic modification or physiological stimulus. Metabonomics has been applied to the study of a variety of diseases and the effects of diet, drugs, toxins, and stress [8]–[12]. A number of analytical tools have been currently employed including ^1^H NMR spectroscopy [13], HPLC/MS [14] and GC/MS [15], [16]. UPLC/MS has enabled better chromatographic peak resolution and increased speed and sensitivity to be obtained for complex mixture separation. It has been considered to have a brighter future in the research of metabonomics.

The toxic effects of ricinine on the metabolic profiles of rats based UPLC/MS was studied in this paper. Principal component analysis (PCA) of the chromatographic data was used to identify the control and the dosed rats based on the differences of their metabolic profiles. Biomarkers associated with the renal damage were determined. Furthermore, histopathology and clinical chemistry studies were also used to confirm the success of hepatic injury.

## Materials and Methods

### Ethics statement

This study was carried out in strict accordance with the recommendations in the Guide for the Care and Use of Laboratory Animals of the National Institutes of Health. The protocol was approved by the Committee on the Ethics of Animal Experiments of the University of China (Permit Number: SYXK 2003-0013).

### Chemicals, reagents, and herbal material

Acetonitrile (HPLC grade) was purchased from Tedia (Fairfield, OH, USA). Formic acid (HPLC grade) was obtained from Dikma Corp (Richmond Hill, NY, USA). The reference standards of phenylalanine, tryptophan, and phosphatidylcholines (38∶6 PC, 36∶4 PC), lysophosphatidylcholines (C18∶2 LPC, C20∶4 LPC, C0∶0/16∶0 LPC, C16∶0/0∶0 LPC, C18∶1/0∶0 LPC, C18∶0/0∶0 LPC) were supplied by Sigma Corporation (St. Louis, MO, USA). Water was purified by redistillation and filtered through 0.22 µm membrane filter before use. Castor seed were purchased from Liaoning Chinese Herbal Medicine Factory (Shenyang, China).

### Herbal material processing

The crude drug was extracted 4 times by refluxing with chloroform (1∶20, w/v) for 4 h each time. The solution obtained was concentrated under reduced pressure until a yellow sticky paste, which was washed 3 times by petroleum ether to remove grease, tannins and so on. Then the yellow residue was recrystallized from anhydrous ethanol under 72°C to obtain ricinine crystal, dried and stored at room temperature until it was administered to rats. The ricinine was indentified by ^1^H-NMR (Bruker Biospin, Germany).

### Animals and treatments

A total of 16 male Wistar rats (180 g–220 g) were provided by Experimental Animal Center of China Medical University (Shenyang, China). The rats were maintained under environmentally controlled breeding room (temperature of 21±2°C, relative humidity of 55±10%, and 12 h/12 h light/dark cycle). They were fed freely available with food and water and housed individually in metabolism cages after acclimation for one week. After that they were separated randomly into four groups (*n* = 6). Three subgroups within the treated group were given ricinine orally twice daily with dosages of 15 mg/kg, 30 mg/kg and 60 mg/kg respectively. The normal control group was given the same volume of water by oral administration. The oral treatment process was proceeded for two weeks continuously.

### Sample collection and preparation

The blood was collected in heparinized tubes for each day from one day before dosing to the end of the experiment. These samples were obtained from the retro-orbital venous plexus and immediately centrifuged at 13 000 rpm for 5 min. The serum was transferred into clean tubes and stored at −80°C until analysis. Prior to the analysis, the serum samples were thawed at room temperature and aliquots of 200 µL were mixed with 200 µL of acetonitrile. The mixture was vortexed for 60 s and centrifuged at 13000 rpm for 10 min. The supernatant was dried by Bath Nitrogen Blow Instrument (L-128B, Beijing, China), reconstituted in 100 µL of the mixture of acetonitrile and water (10∶90, v/v) and vortexed for 60 s, then centrifuged at 13000 rpm for 5 min, and the supernatant was injected for UPLC–MS analysis.

### Serum biochemistry

The blood was collected in different time-points before and after the administration of ricinine to test for hepatic function. These samples were obtained from the retro-orbital venous plexus. Alanine aminotransferase (ALT) and aspartate aminotransferase (AST) were determined for the evaluation of hepatotoxicity disorders.

### Histopathology

The livers were collected immediately after the rats were killed on the last day of the experiment. After fixed in 10% neutral-buffered formalin solution for at least 24 h and dehydrated, embedded in paraffin, the hepatic samples were cutted into 5 µm wax sections. Tissue sections were subsequently stained with hematoxylin–eosin (H–E) for light microscope examination.

### UPLC/MS analysis of serum samples

Rat serum metabolite profiling was performed on a Waters ACQUITY ultra performance liquid chromatography (UPLC) system (Waters Corp., Milford, USA). Chromatographic separation was achieved on a 100 mm×2.1 mm–1.7 µm C18 column (Waters Corp., Milford, MA, USA) with temperature maintained at 40°C. The UPLC mobile phase consisted of 0.1% formic acid in water (solution A) and 0.1% formic acid in acetonitrile (solution B), which was pumped at the flow rate of 0.25 mL/min without split. The gradient elution program is shown in Table 1. All the samples were maintained at 4°C during the analysis.

**Table 1:** Gradient elution program of serum metabolite profiles by UPLC/MS.

Time (min)	Flow (mL/min)	A (%)	B (%)
0	0.25	100	0
0.5	0.25	100	0
20	0.25	5	95
21	0.25	5	95
22	0.25	100	0
25	0.25	100	0

A: water (0.1% formic acid), B: acetonitrile (0.1% formic acid).

Mass spectrometry was performed on a Micromass Quattro Micro API mass spectrometer (Waters Corp., Milford, MA, USA) equipped with an electrospray ionization interface and triple quadrupole mass analyzer in both positive and negative modes using full scan (m/z 110–1000). The following parameters were employed: source temperature of 120°C and desolvation temperature of 350°C, capillary voltage of 3.0 kV and 2.8 kV for positive and negative ionization mode respectively, cone voltage of 35 V for both positive ionization mode negative ionization mode. Nitrogen was used as the desolvation and cone gas, with the flow rate of 400 and 30 L/h, respectively. In the MS/MS experiments, Argon was used as the collision gas and the collision energy was altered between 5 and 25 eV. The mass was corrected with NaCsI before the study. The data were collected in centroid mode.

### Method validation

For method validation study [17], a quality control (QC) sample was prepared by pooling equal volumes (100 µL) of serum from all the analytes that would be encountered during the analysis. The system stability was evaluated by five replicates analysis of QC samples. The precision of the injection was measured with five successive injections of QC sample and the post-preparative stability of sample was evaluated by analyzing five prepared QC samples left at autosampler (maintained at 4°C) for 0, 4, 8, 12 and 24 h. The method repeatability was tested by analysis of five replicates of QC samples.

### Data analysis

The mass data files were processed using the Micromass MarkerLynx Applications Manager version 4.0 (Waters Corp., Milford, USA). This applications manager incorporates a peak deconvolution package that allows detection of the retention time, mass, and intensity of the peaks eluting in each chromatogram. The area of each peak, after being recognized and aligned, was normalized to the summed total ion intensity per chromatogram. The resulting three-dimensional data, peak number (t_R__m/z pair), sample name and normalized ion intensity were introduced to SIMCA-P 11.5 software package (Umetrics, Umea, Sweden) for principal component analysis (PCA). Mean-centered was used for data scaling and centering. ANOVA was performed in succession to reveal the statistical differences for the variables between normal control group and model group. The homogeneity of the variance was tested before ANOVA analysis. Statistical analysis was performed using SPSS (version 19.0; Beijing Stats Data Mining Co. Ltd., China) and one-way ANOVA or Student's t test. P-values less than 0.05 were considered significant and values less than 0.01 were considered highly significant.

### Identification of biomarkers

The potential biomarkers were indentified by available biochemical databases. The following databases have been used: HMDB (http://www.hmdb.ca/), METLIN (http://metlin.scripps.edu/), ChemSpider (http://www.chemspider.com/), KEGG (http://www.kegg.com/), and LIPID MAPS (http://www.lipidmaps.org/).

## Results and Discussion

### Analysis of ricinine by ^1^H-NMR

The ricinine was indentified by ^1^H-NMR (Bruker Biospin, Germany). ^1^H-NMR (300 Hz, DMSO): δ8.10 (1H, d, J = 7.8 Hz, 6-H), 6.43 (1H, dd, J = 7.8 Hz and J = 1.8 Hz, 5-H), 3.98 (3H, s, OCH_3_), 3.43 (3H, s, CH_3_). The results are consistent with the literature.

### Clinical chemistry results

**Figure 1:**
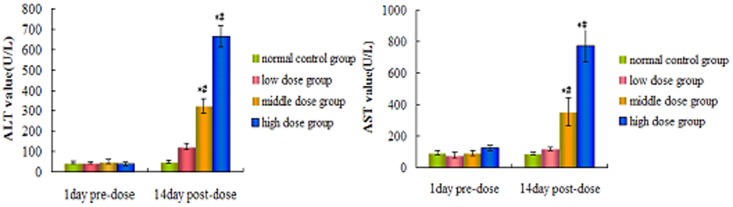
The value of ALT and AST treated with ricinine.

Several clinical serum biochemistry results were measured to monitor the toxic effects of ricinine. Compared with the pre-dose and control groups, the serum ALT and AST concentrations of the low dose groups did not significant difference, but which were elevated significantly (p<0.05) in the middle and high dose groups in 2nd week (Figure 1).

### Histopathology

**Figure 2:**
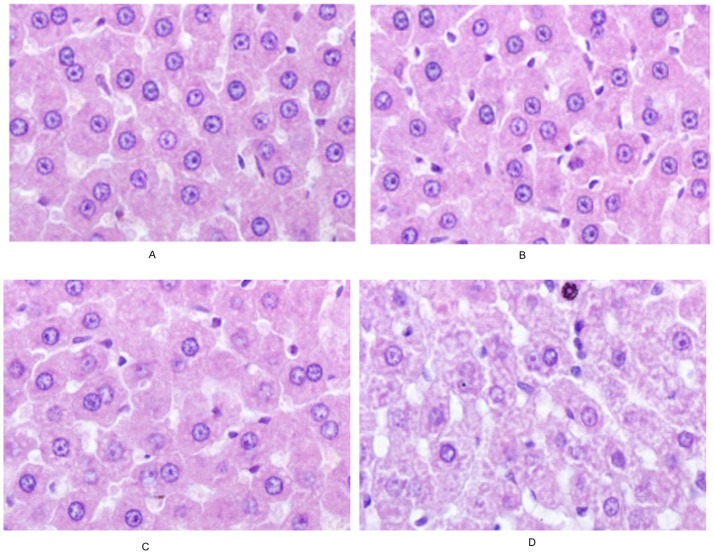
Photomicrographs of liver tissues (HE×200).

The histopathology of hepar exposed to ricinine for 2 weeks was examined. As demonstrated in Figure 2, the hepatocytes were normal in the control group and low dose group. Whereas, the hepatocytes were in vacuolar degeneration and some nucleus were dissolved in the middle dose group. The hepar sections from high dose group showed extensive hepatocyte necrosis and vacuolar degeneration.

### Validation of UPLC–MS conditions

**Figure 3:**
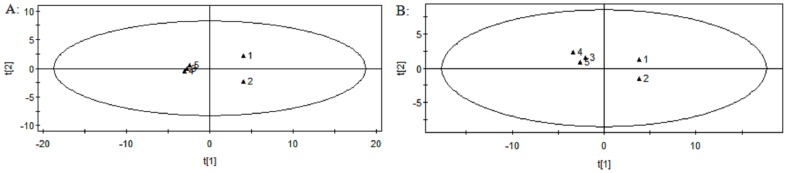
System equilibration test using PCA analysis based on the serum metabolic profiling of QC samples in positive ion mode (A) and negative ion mode (B).

Extracted ion chromatographic peaks of 14 ions were selected according to their chemical polarities and m/z values. The paired retention m/z-time of these ions are 117.1254_1.0, 136.2317_3.0, 146.2154_4.3, 218.1064_7.8, 353.2543_10.4, 524.1346_17.6, 568.4082_14.8 in positive ion mode and 215.1043_1.0, 203.2172_4.2, 201.1363_7.1, 581.1004_9.2, 408.2213_11.4, 564.4331_14.9, 851.1042_20.7 in negative ion mode with retention times covering the whole analytical time. PCA analysis was applied to the system stability. The first two injection samples were separated from the other three samples, while the latter were tight clustered, which gives an indication of the reliability of the data (shown in Figure 3). The precision of injection, within-day stability and repeatability of sample preparation (RSD %) of retention times and peak areas of 14 selected ions in pooled serum samples were calculated (Table 2 and Table 3). The results of precision, stability, and repeatability indicated that the method could be utilized to the analysis of serum samples.

**Table 2:** Precision of injection, within-day stability, repeatability of sample preparation of the serum analytical method in positive ion mode (n = 5).

m/z_t_R_	Precision of injection		Within-day stability		Repeatability of sample prepartion	
	t_R_(RSD%)	Intensity (RSD%)	t_R_(RSD%)	Intensity (RSD%)	t_R_(RSD%)	Intensity (RSD%)
117.1254_1.0	2.3	12.9	3.3	7.9	1.1	8.5
136.2317_3.0	0.3	8.5	0.2	6.4	0.2	9.4
146.2154_4.3	0.5	8.6	0.6	3.1	0.2	8.4
218.1064_7.8	0.9	7.1	0.8	6.8	0.5	5.6
353.2543_10.4	2.4	12.3	0.2	0.2	0.2	8.9
524.1346_17.6	2.1	7.6	3.1	8.9	0.2	6.6
568.4082_14.8	0.6	8.1	0.9	10.1	0.9	7.8

**Table 3:** Precision of injection, within-day stability, repeatability of sample preparation of the serum analytical method in negative ion mode (n = 5).

m/z_t_R_	Precision of injection		Within-day stability		Repeatability of sample prepartion	
	t_R_(RSD%)	Intensity (RSD%)	t_R_(RSD%)	Intensity (RSD%)	t_R_(RSD%)	Intensity (RSD%)
215.1043_1.0	3.7	11.2	2.6	9.5	1.4	5.2
203.2172_4.2	0.6	4.3	0.9	6.5	1.2	3.4
201.1363_7.1	1.5	7.6	2.1	4.6	3.1	2.5
581.1004_9.2	2.6	5.4	1.8	3.7	2.4	2.9
408.213_11.4	1.2	6.9	0.5	2.8	2.3	3.6
564.4331_14.9	0.6	8.1	0.9	1.9	1.4	7.5
851.1042_20.7	0.5	9.6	1.2	4.1	3.4	8.6

### Data analysis

**Figure 4:**
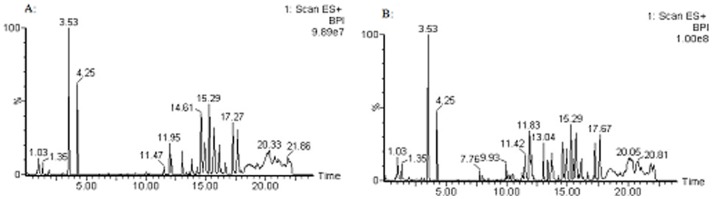
Typical UPLC/MS BPI chromatograms of rat serum metabolite profiles in positive ion mode. A: control group, B: high dose group.

**Figure 5:**
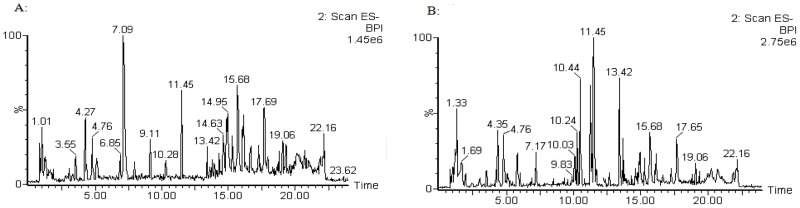
Typical UPLC/MS BPI chromatograms of rat serum metabolite profiles in negative ion mode. A: control group, B: high dose group.

**Figure 6:**
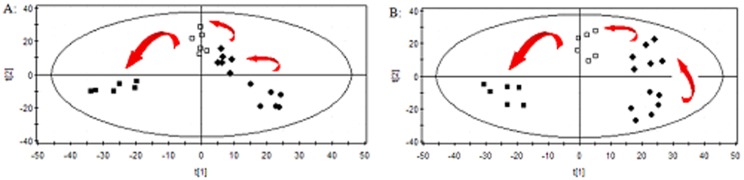
PCA score plots in (A) positive ion mode and in (B) negative ion mode based on the serum metabolic profiling of various dose for rats administrated with ricinine.

The typical positive and negative ion base peak intensity (BPI) chromatograms of a rat serum sample from the normal control group and high dose group were showed in Figure 4 and Figure 5. Although some differences could be visually noted among the sets of detail lustrated in BPI, more subtle changes could be found using a pattern recognition approach, such as PCA. PCA is a chemometric model which reduces matrix of data to their lowest dimension of the most significant factors, was used to detect subtle metabolic changes after ricinine administration. The PCA score plot based on the serum metabolic profiles in different dose-points was showed in Figure 6. At the dose-points of normal control group, low dose group, middle dose group and high dose group, the PCA score plots could be readily divided into four distinct clusters in both positive and negative ion modes, which indicated serum metabolic pattern significantly changed after ricinine administration.

**Figure 7:**
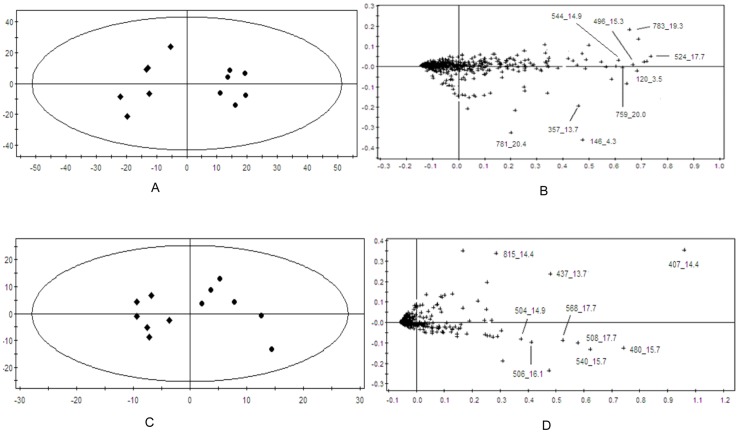
PCA score plots in positive ion mode (A), in negative ion mode (C) and corresponding loading plots in positive ion mode (B), in negative ion mode (D) based on the serum metabolic profiling of 15 mg·kg^−1^ dose for rats administrated with ricinine.

**Figure 8:**
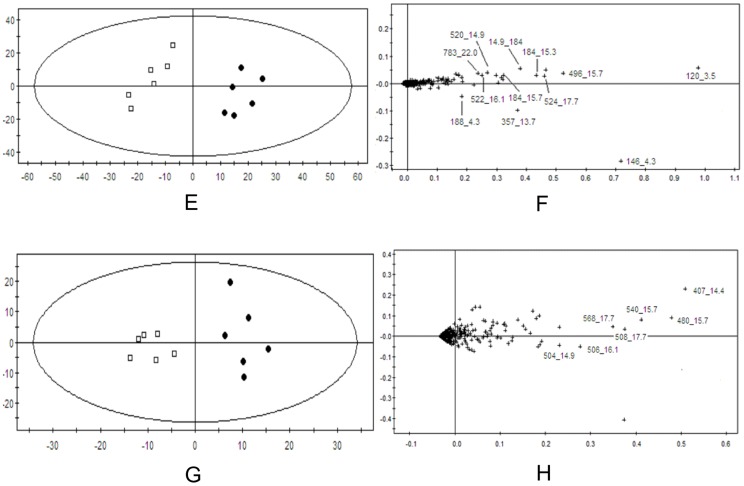
PCA score plots in positive ion mode (E), in negative ion mode (G) and corresponding loading plots in positive ion mode (F), in negative ion mode (H) based on the serum metabolic profiling of 15 mg·kg^−1^ dose for rats administrated with ricinine.

**Figure 9:**
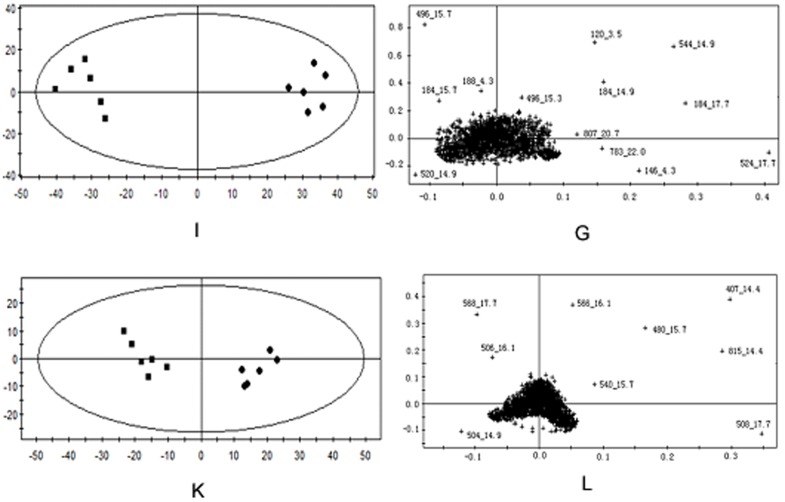
PCA score plots in positive ion mode (I), in negative ion mode (K) and corresponding loading plots in positive ion mode (J), in negative ion mode (L) based on the serum metabolic profiling of 15 mg·kg^−1^ dose for rats administrated with ricinine.

In order to evaluate dose-dependent effect of ricinine on the serum metabolic pattern, a PCA model was constructed to analyze all the data acquired from model group and treatment group. At the different dose-points of 15 mg·kg^−1^ (Figure 7), 30 mg·kg^−1^ (Figure 8), and 60 mg·kg^−1^ (Figure 9) in positive and negative modes, the PCA score plots could be readily divided into two clusters, which indicated serum metabolic pattern significantly changed after the treatment of ricinine.

### Biomarker identification

**Figure 10:**
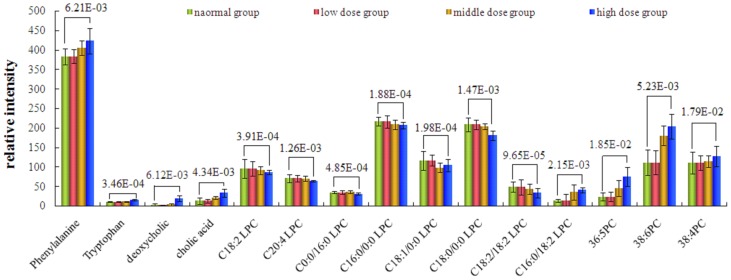
Comparison of the relative intensity of putative potential biomarkers in the plasma of the control group and ricinine-treated group.

The loading profiles (Figure 7, Figure 8 and Figure 9) that visualizes the influences of variables, was used for the selection of biomarkers. Judged by the distance from the origin, a series of ions which were found predominantly in the loading plot were chosen as biomarkers (Table 4). Based on the relative intensities of the metabolites from the normalized spectrum, ANOVA was used to reveal the significant differences of identified metabolites between the control group and treated group. The significant variables were showed gradual rising trend along with dose and were summarized in Figure 10.

**Table 4:** Potential biomarkers of control group and treated group based on serum metabolite profiles detected by UPLC/MS.

t_R_(min)	m/z(Da)	Scan mode	Quasi-molecular ion	Identification	Change trend compared with the control	Fold change compared with the control
3.5	119.7486	+	[M+H−HCOOH]^+^	phenylalanine	↑	1.18
4.3	146.0154	+	[M−NH_3_−HCOOH+H]^+^	tryptophan	↑	1.71
	188.0432	+	[M−NH_3_+H]^+^			
13.7	357.4052	+	[M+H−2H20]+	deoxycholic acid	↑	4.02
	437.421	−	[M+HCOO]^−^			
14.4	407.1061	−	[M−H]^−^	cholic acid	↑	2.24
	815.1432	−	[2M−H]^−^			
14.9	184.0064	+	[H_2_O_3_PO−CH_2_CH_2_N(CH_3_)_3_]^+^	C18∶2 LPC	↓	0.95
	504.2021	−	[M−CH_3_]^−^			
	520.2123	+	[M+H]^+^			
14.9	544.0104	+	[M+H]^+^	C20∶4 LPC	↓	0.86
15.3	184. 0064	+	[H_2_O_3_PO−CH_2_CH_2_N(CH_3_)_3_]^+^	C0∶0/16∶0 LPC	↓	0.45
	496.2211	+	[M+H]^+^			
15.7	184. 0064	+	[H_2_O_3_PO−CH_2_CH_2_N(CH_3_)_3_]^+^	C16∶0/0∶0 LPC	↓	0.95
	480.2026	−	[M−CH_3_]^−^			
	496.2211	+	[M+H]^+^			
	540.3012	−	[M+HCOO]^−^			
16.1	184.0064		[H_2_O_3_PO−CH_2_CH_2_N(CH_3_)_3_]^+^	C18∶1/0∶0 LPC	↓	0.88
	522.2001	+	[M+H]^+^			
	506.1342	−	[M−CH_3_]^−^			
17.7	184.0064	+	[H_2_O_3_PO−CH_2_CH_2_N(CH_3_)_3_]^+^	C18∶0/0∶0 LPC	↓	0.85
	508.2031	−	[M−CH_3_]^−^			
	524.1346	+	[M+H]^+^			
	568.4082	−	[M+HCOO]^−^			
19.3	783.1213	+	[M+H]^+^	PC 18∶2/18∶2	↓	0.61
20	759.1042	+	[M+H]^+^	PC 16∶0/18∶2	↑	3.03
20.4	781.1432	+	[M+H]^+^	36∶5 PC	↑	3.75
20.7	809.972	+	[M+H]^+^	38∶6 PC	↑	1.83
22	783.2213	+	[M+H]^+^	36∶4 PC	↑	1.14

* The identified metabolites were confirmed by standards (see Supporting Information).

**Figure 11:**
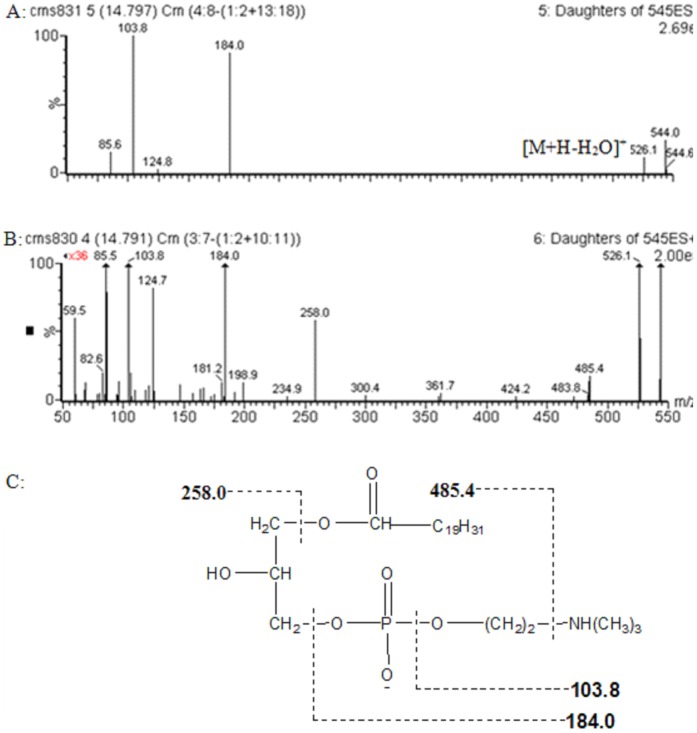
product ion spectrum of m/z 544 in positive mode and possible MS fragmentation mechanism.

The identification of the biomarkers was performed on a comparison of their MS/MS spectra and retention time with those of commercially available standards, data in the literature, and database resources. The related pathway of each biomarker was also given by searching HMDB, KEGG database et al. The biomarker with the retention time and m/z pair 14.9_544 in positive ion mode was identified as C20∶4 LPC, which is used as an example to illustrate the identification process. In positive ion spectrum (Figure 11A), besides the base peak ion at m/z 544.0, the ions at m/z 526.1, 184.0 and 103.8 were found. The initial assessment is that the quasimolecular ion is m/z 544.0 ([M+H]^+^) and the ions at m/z 526.1 is the adduction[M+H-H_2_O]^+^. The ions at m/z 184.0 and 103.8 are typical fragment ions of phosphatidylcholine, which are [H_2_O_3_PO-CH_2_CH_2_N(CH_3_)_3_]^+^ and [HOCH_2_CH_2_N(CH_3_)_3_]^+^, respectively. Thus, the ion at m/z 544.0 is phosphatidylcholine was inferred. Then, the MS/MS information about high abundance fragment of m/z 194 was acquired from the high tension scan mode (Figure 11B). The m/z of two major fragment ions were 258.0 and 485.4, which represented the fragments [M+H-OCOC_19_H_31_] and [M+H-NH(CH_3_)_3_], respectively. Finally, it was identified as C20∶4 LPC by comparing with the fragmentation pattern of compound (HMDB10396) in HMDB database. Literature retrieval was also performed [18]. The possible fragment mechanism was deduced (Figure 11C). Finally, standard compounds phenylalanine, tryptophan, phosphatidylcholines (38∶6 PC, 36∶4 PC), lysophosphatidylcholines (C18∶2 LPC, C20∶4 LPC, C0∶0/16∶0 LPC, C16∶0/0∶0 LPC, C18∶1/0∶0 LPC, C18∶0/0∶0 LPC) were used to confirm the identified metabolites, and the deoxycholic acid, cholic acid, phosphatidylcholines (18∶2/18∶2, 16∶0/18∶2, 36∶5) were indentified by biochemical databases. The MS/MS spectra of standards information was available as supporting Information (see Supplementary materials).

### Biochemical interpretation

According to the 14 days after administration of ricinine, PCA scores and loading plots and the histopathology results suggested that hepatic damage was clearly induced by ricinine. The change trends of the metabolites identified are given in Table 4. The results effectively indicated that these metabolites may be the biomarkers of hepatotoxicity effects of ricinine, which were related to the action mechanism of hepatotoxicity. In the ricinine-treated group, phenylalanine, tryptophan, deoxycholic acid, cholic acid, PC 16∶0/18∶2, 36∶5 PC, 38∶6 PC and 36∶4 PC were significantly increased compared with that in the normal control group. However, C18∶2 LPC, C20∶4 LPC, C0∶0/16∶0 LPC, C16∶0/0∶0 LPC, C18∶1/0∶0 LPC, C18∶0/0∶0 LPC and PC 18∶2/18∶2 were significantly decreased. The difference was more and more obvious with the dose increased.

Phenylalanine and tryptophan are essential amino acids, which belongs to aromatic amino acid. Phenylalanine is biologically converted into tyrosine, another one of the DNA-encoded amino acids. Tyrosine in turn is converted into DOPA, which is further converted into dopamine, norepinephrine (noradrenaline), and epinephrine (adrenaline). The latter three are known as the catecholamines. Tryptophan acts as building blocks in protein biosynthesis, which were synthesized 5-hydroxy tryptamine about 1%–2% and most of the rest of the tryptophan metabolise in the liver. It were reported that branched chain amino acid was decreased and aromatic amino acid was increased in hepatotoxicity metabolites [19]–[21]. In this study, increase of phenylalanine and creatinine were observed in serum metabolite profiles of high dose group compared with normal control group, which showed that hepatotoxicity are involved in amino acid metabolism.

Deoxycholic acid and cholic acid is a bile acid, which is often used as potential biomarker in hepatotoxicity [22]. Bile acids are synthesized in the liver and secreted in the gallbladder or in the intestine, conjugated mainly with taurine and glycine. In hepatobiliary and intestinal disease, disturbances of synthesis, metabolism, and clearance by the liver and absorption by the intestine will affect the concentration and profile of bile acids in various pool compartments [23], [24]. In our study, high dose of ricinine caused the elevated level of cholic acid, which showed that hepatotoxicity is involved in enterohepatic circulation.

Phosphatidylcholines (PC) are a major component of biological membranes. Lysophosphatidylcholine (LPC) is the intermediate metabolites of PC. Predominantly polyunsaturated LPC are produced by phospholipase A2 (PLA2) and saturated LPCs are principally resulted from the activity of lecithin: cholesterol acyltransferase (LCAT) [25]–[27]. LPC are biologically active lipids regulating a variety of cellular functions. LPC has been suggested to play a functional role in various diseases, and data indicate a potential use of LPC as a diagnostic marker. However, evidence variation suggests that LPC also exerts direct biological effects, especially on lipometabolism. LPC also promote inflammation and participate in the regulation of autoimmune [28], [29]. In our study, high dose of ricinine caused the elevated level of PC and lessened level of LPC, which showed that hepatotoxicity are involved in lipometabolism and immune system.

## Conclusions

A metabonomics method based on UPLC/MS has been developed to study the toxic effects treated with ricinine. With pattern recognition analysis (PCA), a clear separation of ricinine-treated group and control group was achieved. Some potential biomarkers such as phenylalanine, tryptophan, cholic acid, LPC and PC have been found and identified. Their changes indicated pharmacological effects of ricinine on amino acid metabolism, enterohepatic circulation, lipometabolism and immune system. This study indicates that UPLC/MS-based metabonomic analysis is useful for predicting the hepatotoxicity induced by ricinine.

## Supporting Information

Figure S1 **The MS/MS spectrum of phenylalanine.**
(TIF)

Figure S2 **The MS/MS spectrum of tryptophan.**
(TIF)

Figure S3 **The MS/MS spectrum of C18∶2 LPC, A: ES+, B: ES−.**
(TIF)

Figure S4 **The MS/MS spectrum of C20∶4 LPC.**
(TIF)

Figure S5 **The MS/MS spectrum of C0∶0/16∶0 LPC.**
(TIF)

Figure S6 **The MS/MS spectrum of C16∶0/0∶0 LPC. A: ES+, B: ES−.**
(TIF)

Figure S7 **The MS/MS spectrum of C18∶1/0∶0 LPC. A: ES+, B: ES−.**
(TIF)

Figure S8 **The MS/MS spectrum of C18∶0/0∶0 LPC.**
(TIF)

Figure S9 **The Ms Spectrum of 36∶4PC.**
(TIF)

Figure S10 **The Ms Spectrum of 38∶6PC.**
(TIF)

## References

[1] National Pharmacopoeia Committee (2010) *Ricinus communis*. Pharmacopoeia of People's Republic of China. Part 1. Chemical Industry Press, Beijing: pp330.

[2] Ferraz AC, Angelucci ME, Da Costa ML, Batista IR, De Oliveira BH, et al (1999) Pharmacological evaluation of ricinine, a central nervous system stimulant isolated from Ricinus communis. Pharmacol Biochem Behav 63: 367–375. 10418776 10.1016/s0091-3057(99)00007-6

[3] Gandhi VM, Cherian KM, Mulky MJ (1994) Detoxification of castor seed meal by interaction with sal seed meal. JOACS 71: 827–831.

[4] Bradberry SM, Dickers KJ, Rice P, Griffiths GD, Vale JA (2003) Ricin poisoning. Toxicol Rev 22: 65–70. 14579548 10.2165/00139709-200322010-00007

[5] Kang S, Cordell G, Soejarto D, Fong H (1985) Alkaloids and flavoids from Ricinus communis. J Nat Prod 48: 155–156.

[6] Worbs S, Köhler K, Pauly D, Avondet MA, Schaer M, et al (2011) Ricinus communis intoxications in human and veterinary medicine-a summary of real cases. Toxins (Basel) 3: 1332–72. 22069699 10.3390/toxins3101332 PMC3210461

[7] Robinson T (1987) Precursors of ricinine in the castor bean plant. Biochem 17: 1903–1905.

[8] Lindon JC, Keun HC, Ebbels TM, Pearce JM, Holmes E, et al (2005) The Consortium for Metabonomic Toxicology (COMET): aims, activities and achievements. Pharmacogenomics 6: 691–699. 16207146 10.2217/14622416.6.7.691

[9] Lafaye A, Junot C, Ramounet-Le Gall B, Fritsch P, et al (2003) Metabolite profiling in rat urine by liquid chromatography/electrospray ion trap mass spectrometry. Application to the study of heavy metal toxicity. Rapid Commun Mass Spectrom 17: 2541–2549. 14608626 10.1002/rcm.1243

[10] Brindle JT, Nicholson JK, Schofield PM, Grainger DJ, Holmes E (2003) Application of chemometrics to 1H NMR spectroscopic data to investigate a relationship between human serum metabolic profiles and hypertension. Analyst 128: 32–36. 12572799 10.1039/b209155k

[11] Griffin JL, Bonney SA, Mann C, Hebbachi AM, Gibbons GF, et al (2004) An integrated reverse functional genomic and metabolic approach to understanding orotic acid-induced fatty liver. Physiol Genomics 17: 140–149. 14747661 10.1152/physiolgenomics.00158.2003

[12] Brindle JT, Antti H, Holmes E, Tranter G, Nicholson JK, et al (2002) Rapid and noninvasive diagnosis of the presence and severity of coronary heart disease using 1H-NMR-based metabonomics. Nat Med 8: 1439–1444. 12447357 10.1038/nm1202-802

[13] Hasim A, Ma H, Mamtimin B, Abudula A, Niyaz M, et al (2012) Revealing the metabonomic variation of EC using 1H-NMR spectroscopy and its association with the clinicopathological characteristics. Mol Biol Rep 39: 8955–8964. 22736106 10.1007/s11033-012-1764-z

[14] Wang J, Reijmers T, Chen L, Van Der Heijden R, Wang M, et al (2009) Systems toxicology study of doxorubicin on rats using ultra performance liquid chromatography coupled with mass spectrometry based metabolomics. Metabolonics 5: 407–418. 10.1007/s11306-009-0165-3 PMC2794350 20046867

[15] Guallar-Hoyas C, Turner MA, Blackburn GJ, Wilson ID, Thomas CL (2012) A workflow for the metabolomic/metabonomic investigation of exhaled breath using thermal desorption GC-MS. Bioanalysis 4: 2227–2237. 23046265 10.4155/bio.12.193

[16] Dunn WB, Broadhurst D, Ellis DI, Brown M, Halsall A, et al (2008) A GC-TOF-MS study of the stability of serum and urine metabolomes during the UK Biobank sample collection and preparation protocols. Int J Epidemiol 37 Suppl 1: i23–30. 18381390 10.1093/ije/dym281

[17] Gika Helen G, Theodoridis Georgios A, Wingate Julia E, Wilson Ian D (2007) Within-Day Reproducibility of an HPLC-MS-Based Method for Metabonomic Analysis: Application to Human Urine. J Proteome Research 6: 3291–3303. 17625818 10.1021/pr070183p

[18] Takatera A, Takeuchi A, Saiki K, Morisawa T (2006) Quantification of lysophosphatidylcholines and phosphatidylcholines using liquid chromatography-tandem mass spectrometry in neonatal serum. J Chromatography B 838: 31–36. 10.1016/j.jchromb.2006.03.006 16603422

[19] Limberg B, Kommerell B (1984) Correction of altered plasma amino acid pattern in cirrhosis of the liver by somatostatin. Gut 25: 1291–1295. 6149982 10.1136/gut.25.11.1291 PMC1432315

[20] Montanari A, Simoni I, Vallisa D, Trifirò A, Colla R, et al (1988) Free amino acids in plasma and skeletal muscle of patients with liver cirrhosis. Hepatology 8: 1034–1039. 3417224 10.1002/hep.1840080509

[21] Morgan MY, Marshall AW, Milsom JP, Sherlock S (1982) Plasma amino-acid patterns in liver disease. Gut 23: 362–370. 7076013 10.1136/gut.23.5.362 PMC1419690

[22] Li H, Ni Y, Su M, Qiu Y, Zhou M, et al (2007) Pharmacometabonomic phenotyping reveals different responses to xenobiotic intervention in rats. J Proteome Res 6: 1364–1370. 17311441 10.1021/pr060513q

[23] Palmeira CM, Rolo AP (2004) Mitochondrially-mediated toxicity of bile acids. Toxicology 203: 1–15. 15363577 10.1016/j.tox.2004.06.001

[24] Ostrow JD (1993) Hepatic Transport and Bile Secretion: Physiology and Pathophysiology. Raven Press, New York: 673–712.

[25] Sekas G, Patton GM, Lincoln EC, Robins SJ (1985) Origin of plasma lysophosphatidylcholine: evidence for direct hepatic secretion in the rat. J Lab Clin Med 105: 190–194. 3973457

[26] Graham A, Zammit VA, Christie WW, Brindley DN (1991) Sexual dimorphism in the preferential secretion of unsaturated lysophosphatidylcholine by rat hepatocytes but no secretion by sheep hepatocytes. Biochim Biophys Acta 1081: 151–158. 1998732 10.1016/0005-2760(91)90020-i

[27] Brindley DN (1993) Hepatic secretion of lysophosphatidylcholine: a novel transport system for polyunsaturated fatty acids and choline. J Nutr Biochem 4: 442–449.

[28] Fuchs B, Schiller J, Wagner U, Häntzschel H, Arnold K (2005) The phosphatidtlcholine/lysophosphatidylcholine ratio in human plasma is an indicator of the severity of rheumatoid arthritis: investigations by ^31^P NMR and MALDI-TOF MS. Clin Biochem 38: 925–933. 16043165 10.1016/j.clinbiochem.2005.06.006

[29] Kabarowski JH, Zhu K, Le LQ, Witte ON, Xu Y (2001) Lysophosphatidylcholine as a ligand for the immunoregulatory receptor G2A. Science 293 (5530) 702–705. 11474113 10.1126/science.1061781

