# Supplementary figures and images for: A Metabonomic Analysis of Serum from Rats Treated with Ricinine Using Ultra Performance Liquid Chromatography Coupled with Mass Spectrometry
Source: PLoS One. 2014 Mar 11;9(3):e90416. doi: 10.1371/journal.pone.0090416 (PMC3949718; doi:10.1371/journal.pone.0090416)

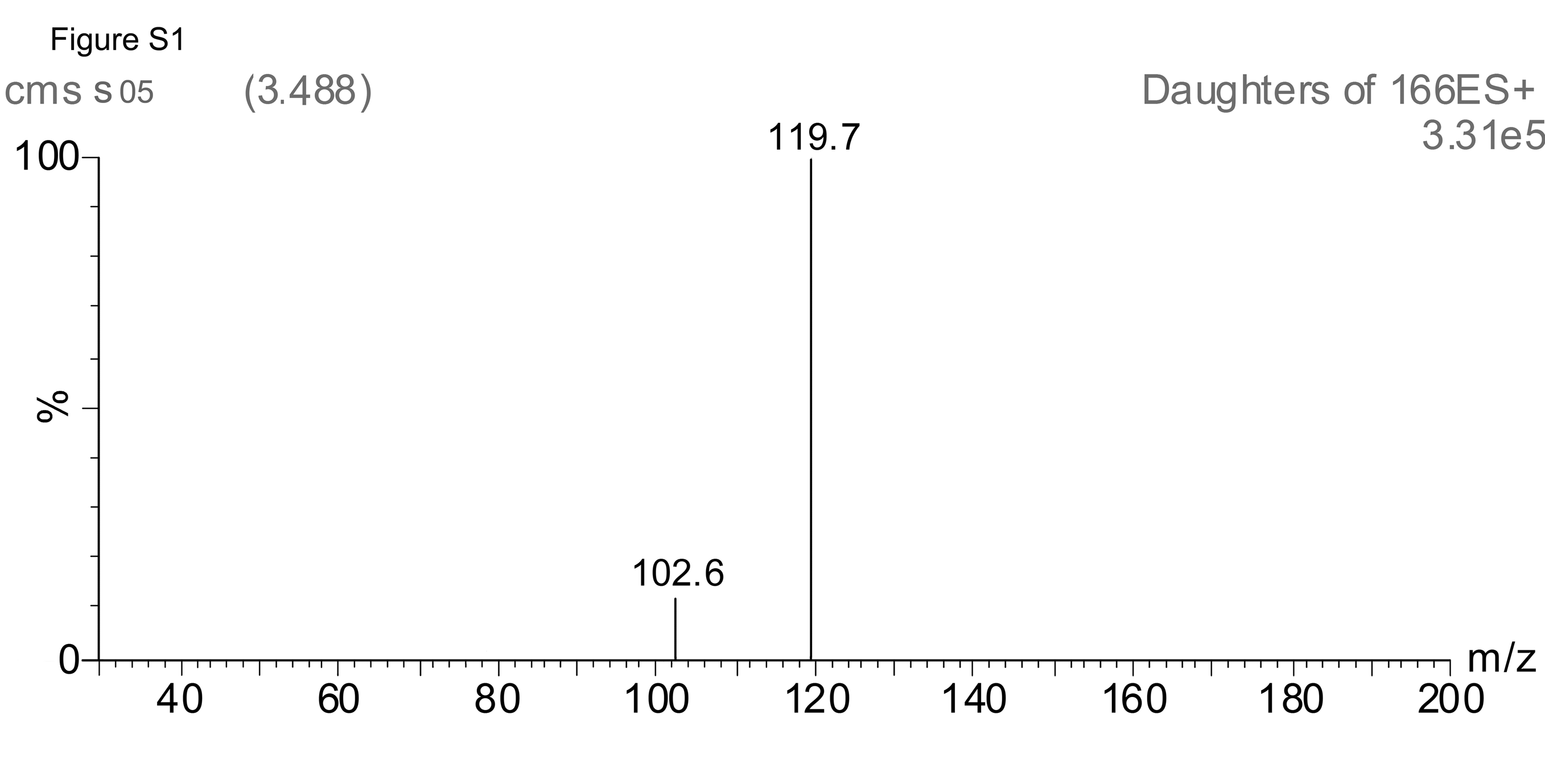

Supplement: Figure S1 — The MS/MS spectrum of phenylalanine. (TIF) [file pone.0090416.s001.tif]

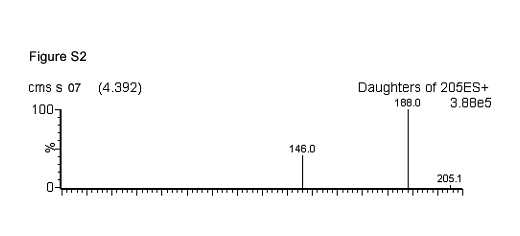

Supplement: Figure S2 — The MS/MS spectrum of tryptophan. (TIF) [file pone.0090416.s002.tif]

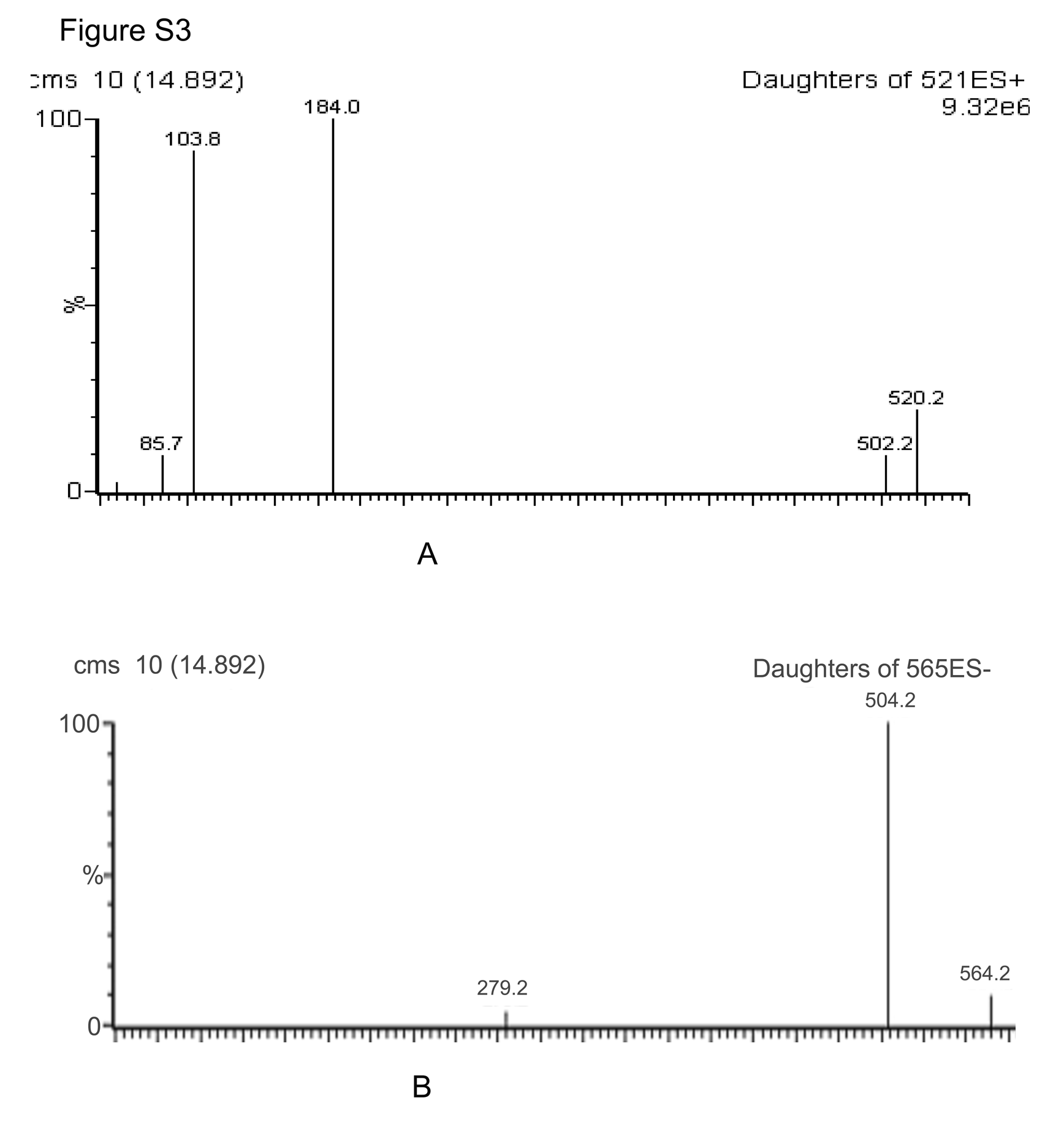

Supplement: Figure S3 — The MS/MS spectrum of C18∶2 LPC, A: ES+, B: ES−. (TIF) [file pone.0090416.s003.tif]

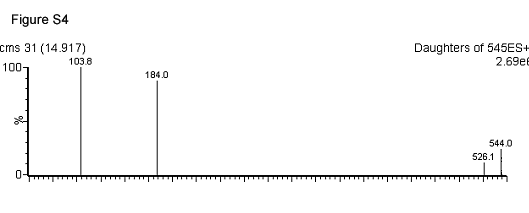

Supplement: Figure S4 — The MS/MS spectrum of C20∶4 LPC. (TIF) [file pone.0090416.s004.tif]

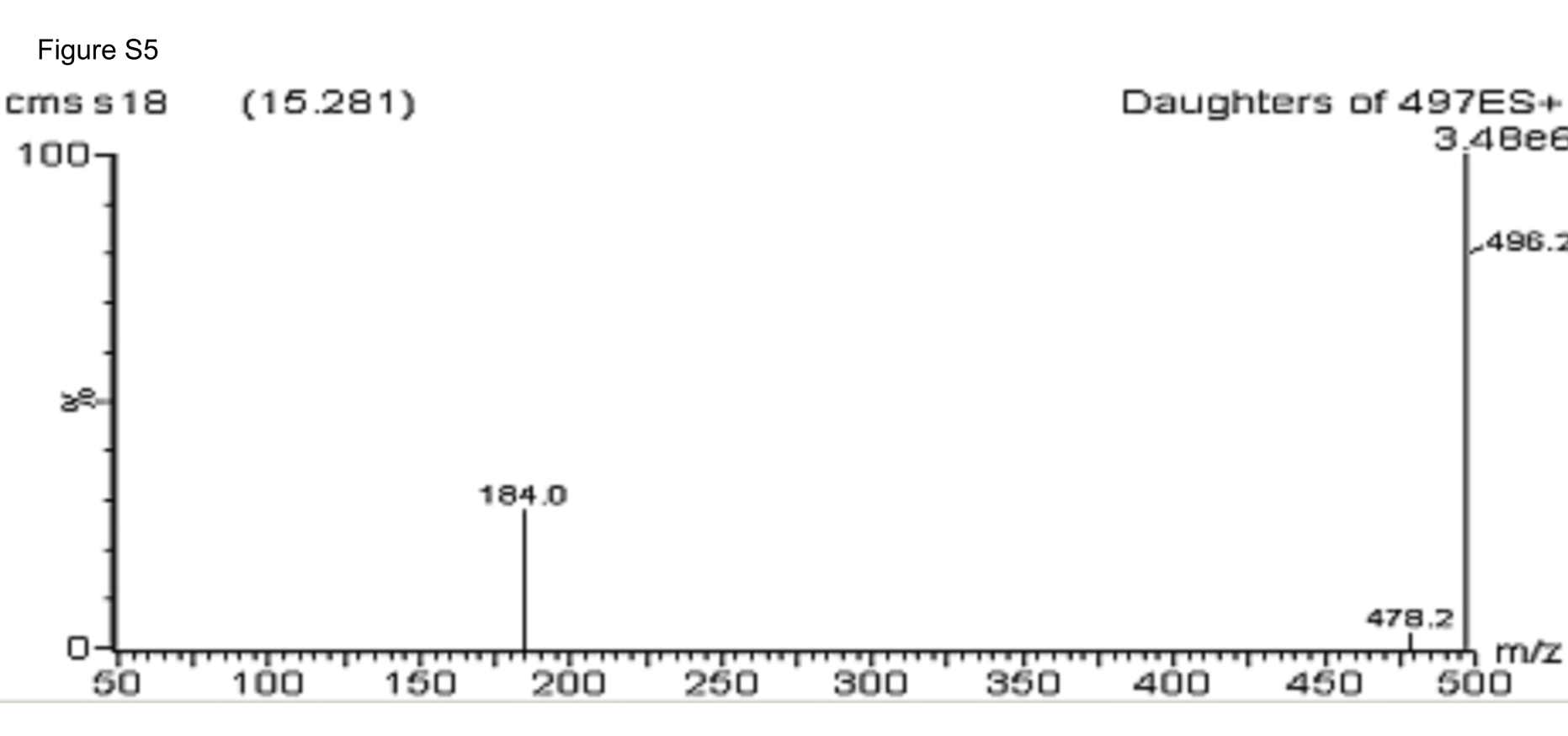

Supplement: Figure S5 — The MS/MS spectrum of C0∶0/16∶0 LPC. (TIF) [file pone.0090416.s005.tif]

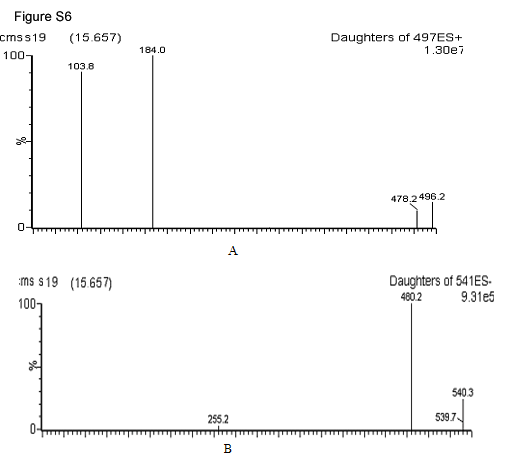

Supplement: Figure S6 — The MS/MS spectrum of C16∶0/0∶0 LPC. A: ES+, B: ES−. (TIF) [file pone.0090416.s006.tif]

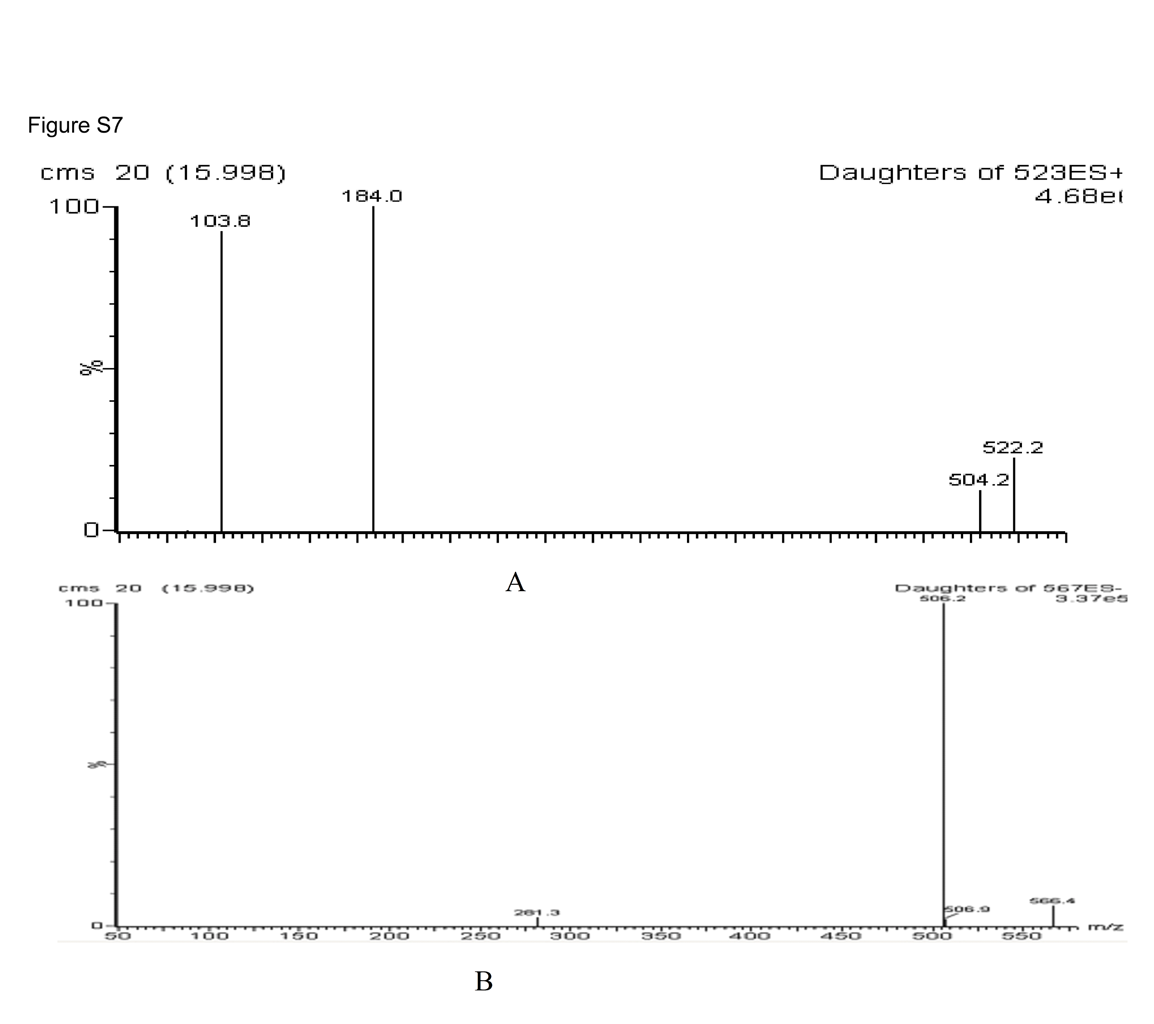

Supplement: Figure S7 — The MS/MS spectrum of C18∶1/0∶0 LPC. A: ES+, B: ES−. (TIF) [file pone.0090416.s007.tif]

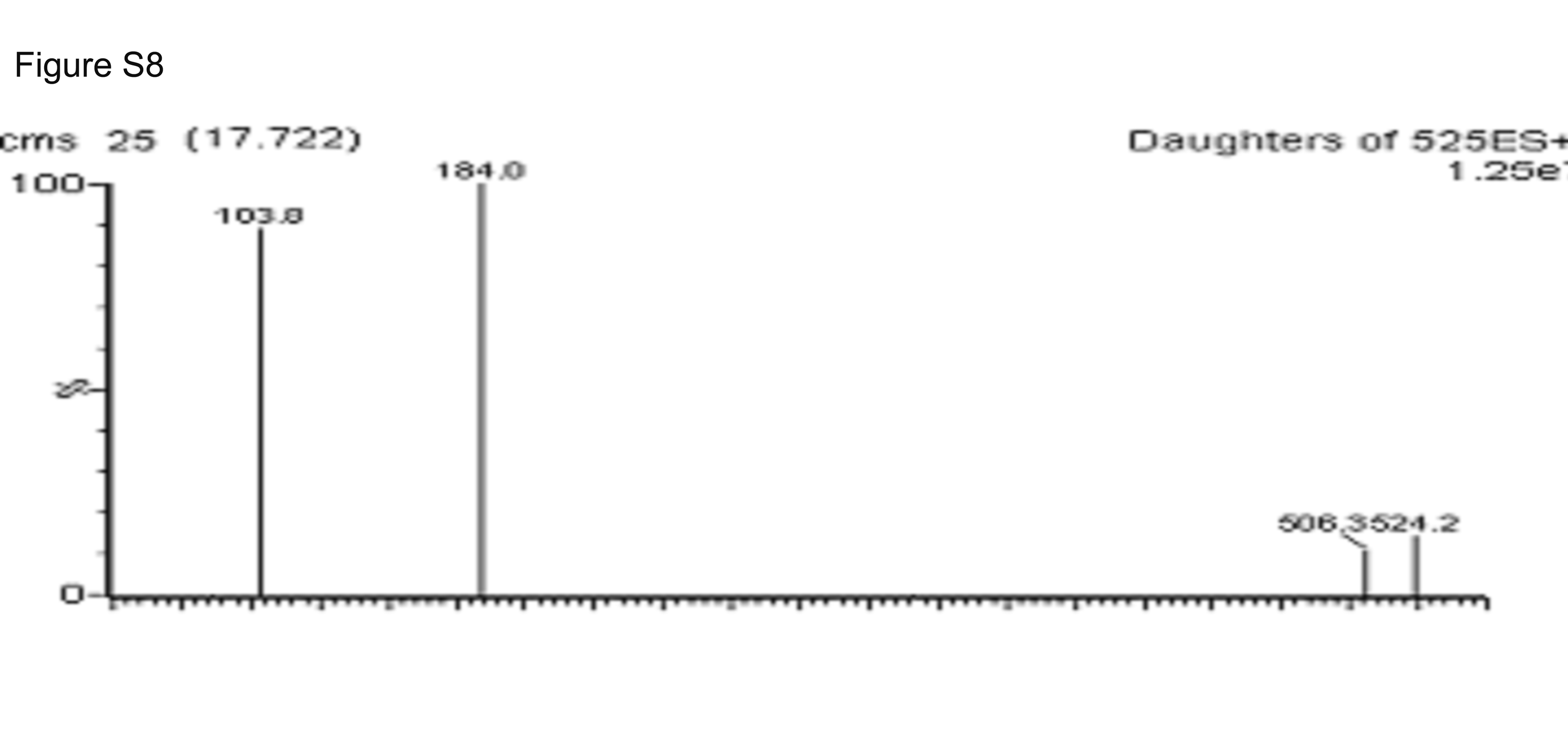

Supplement: Figure S8 — The MS/MS spectrum of C18∶0/0∶0 LPC. (TIF) [file pone.0090416.s008.tif]

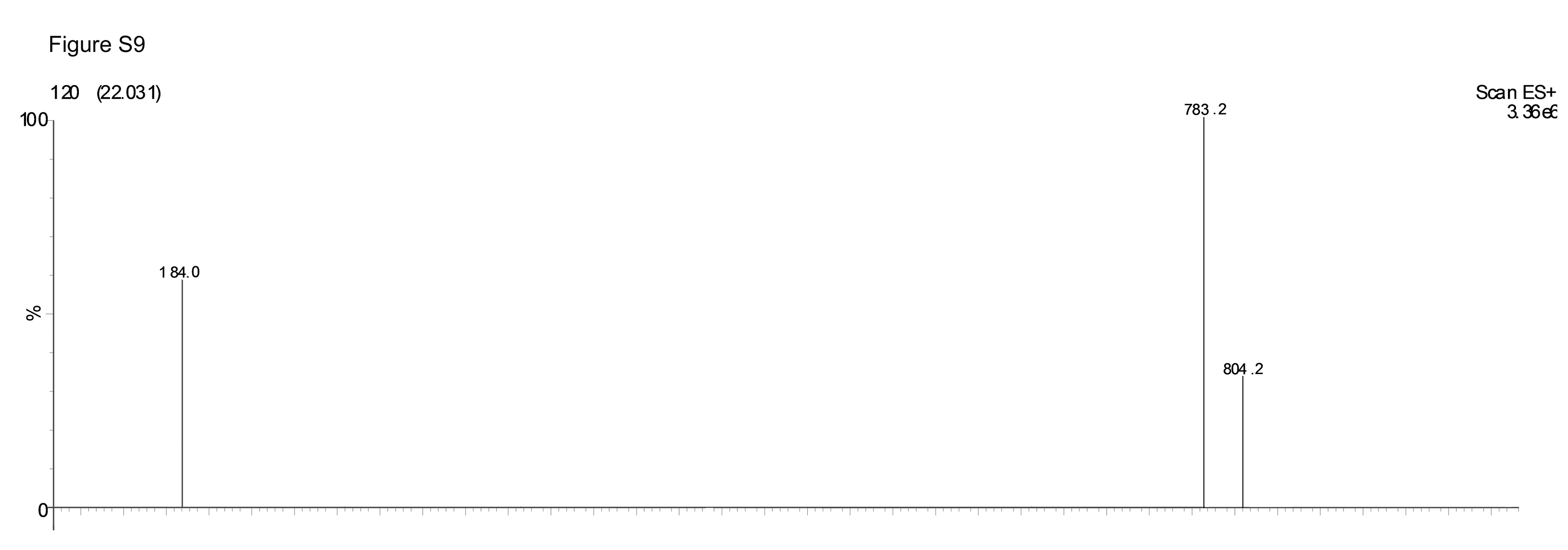

Supplement: Figure S9 — The Ms Spectrum of 36∶4PC. (TIF) [file pone.0090416.s009.tif]

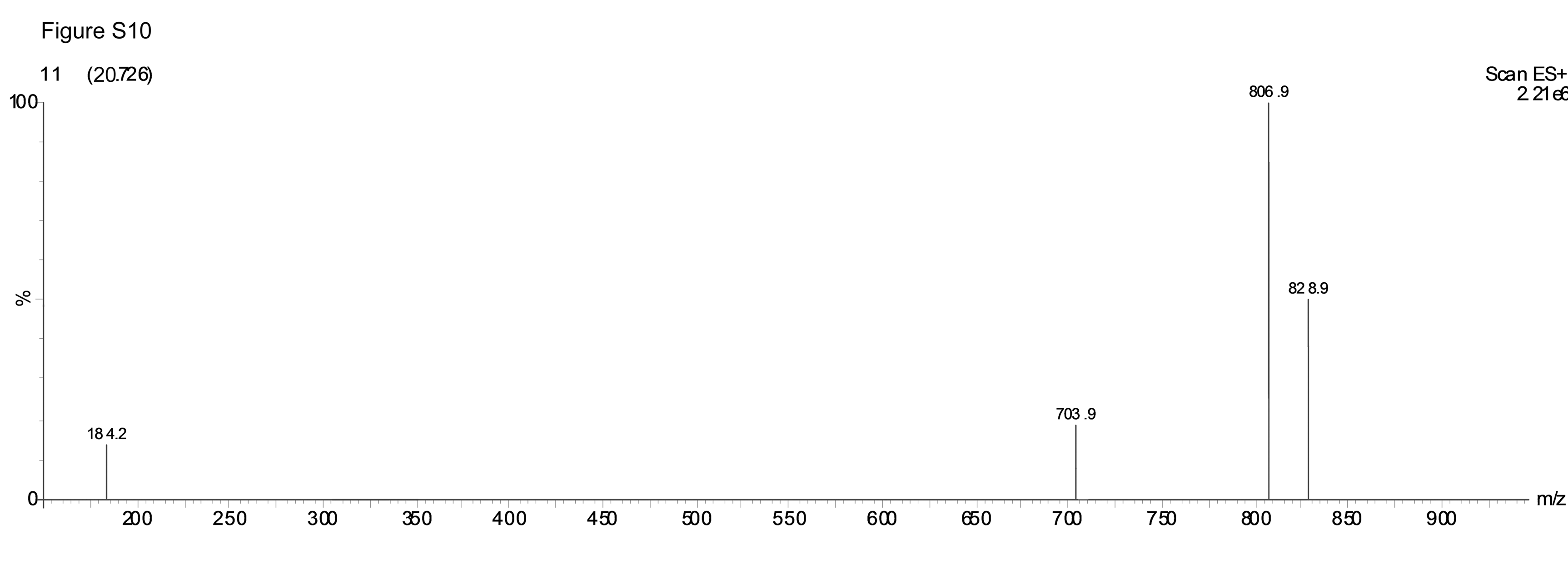

Supplement: Figure S10 — The Ms Spectrum of 38∶6PC. (TIF) [file pone.0090416.s010.tif]
